# Comparison of Three Different Waves in Healthcare Workers during the COVID-19 Pandemic: A Retrospective Observational Study in an Italian University Hospital

**DOI:** 10.3390/jcm11113074

**Published:** 2022-05-29

**Authors:** Luigi De Maria, Stefania Sponselli, Antonio Caputi, Antonella Pipoli, Gianmarco Giannelli, Giuseppe Delvecchio, Silvia Zagaria, Domenica Cavone, Pasquale Stefanizzi, Francesco Paolo Bianchi, Silvio Tafuri, Luigi Vimercati

**Affiliations:** 1 Interdisciplinary Department of Medicine, Section of Occupational Medicine, University of Bari, 70124 Bari, Italy; luigi.demaria@uniba.it (L.D.M.); stefania.sponselli@uniba.it (S.S.); antonio.caputi@uniba.it (A.C.); antonella.pipoli@uniba.it (A.P.); gianm_94@yahoo.it (G.G.); g.delvecchio27@studenti.uniba.it (G.D.); silvia.zagaria@uniba.it (S.Z.); domenica.cavone@uniba.it (D.C.); 2 Occupational Medicine Unit, University Hospital of Bari, 70124 Bari, Italy; 3 Interdisciplinary Department of Medicine, University of Bari, 70124 Bari, Italy; pasqualestef@gmail.com (P.S.); frapabi@gmail.com (F.P.B.); silvio.tafuri@uniba.it (S.T.)

**Keywords:** healthcare workers (HCWs), COVID-19, biological risk

## Abstract

Background: SARS-CoV-2 has infected many healthcare workers and (HCWs) worldwide. The aim of this study was to determine, analyze, and compare the frequency and characteristics of COVID-19 cases among HCWs of the University Hospital of Bari. Methods: A retrospective observational study was conducted after preventive protocol implementation. The SARS-CoV-2 infection frequency was determined by real-time reverse transcription-polymerase chain reaction on nasopharyngeal samples. Results: Overall, 519 HCWs (9%) tested positive among a total of 6030 HCWs during the three waves. The highest frequency of COVID-19 cases (*n* = 326; 63%) was observed during the 2nd wave, from September 2020 to December 2020, and the lowest (*n* = 34; 7%) was observed during the 1st wave, from March 2020 to August 2020 (*p* < 0.001). Working in a designated COVID-19 department was not a risk factor for infection. Conclusions: The correct use of personal protective equipment and the early identification of symptomatic workers are still essential factors to avoid nosocomial clusters, even in this current phase of vaccine availability.

## 1. Introduction

The healthcare system is burdened by the SARS-CoV-2 pandemic. In the first months of 2020, Italy was the most affected country in terms of infections, deaths, and hospitalizations [1]. To date, more than 225,000 cases have been identified among healthcare workers (HCWs) since the start of the pandemic [2]. HCWs play a central role in handling the ongoing coronavirus disease 2019 (COVID-19) pandemic [3,4]. Monitoring HCWs, both symptomatic and asymptomatic, through screening programs is crucial to rapidly identify and isolate infected subjects and, consequently, to avoid hospital infection outbreaks and to allow healthcare workers to return to work promptly [5,6,7,8]. Moreover, alongside screening programs, the implementation of preventive measures and protocols in hospital settings has been shown to be highly effective in reducing the number of cases [9].

At the University Hospital of Bari, Apulia, southern Italy, during the first months of the pandemic (March–April 2020), a preventive protocol was implemented by the Operative Unit of Occupational Medicine according to the latest guidelines published by the World Health Organization (WHO) and the Centers for Disease Control and Prevention (CDC) [10,11]. Only 25 HCWs infected by SARS-CoV-2 were identified from 3109 nasopharyngeal swabs analyzed with real-time reverse transcription-polymerase chain reaction (RT–PCR), despite the high number of patients admitted to the emergency department and hospitalized during the period of observation. However, this result is due to a period of low COVID-19 prevalence in the overall population of southern Italy during a short period of time (first months of the pandemic in Italy) [12,13,14,15].

In the present study, we extended the period of data collection and analyzed molecular swab results during the 2nd and 3rd epidemic waves of COVID-19, which occurred from the autumn of 2020 to the summer of 2021. Therefore, the aim of this study was to determine, analyze, and compare the frequency and characteristics of COVID-19 cases among HCWs of the University Hospital of Bari, one of the major COVID-19 hub centers in southern Italy, during three different observation periods from March 2020 to August 2021, defined as “waves”.

## 2. Methods

### 2.1. Study Design, Setting, and Population

We conducted a retrospective observational study at the University Hospital of Bari, southern Italy, during the SARS-CoV-2 pandemic on a population of 6030 HCWs. We collected the occupational characteristics of all HCWs who underwent nasopharyngeal swab RT–PCR tests (NSTs) for the detection of a SARS-CoV-2 infection in the three periods defined as “waves”: the 1st wave (from 12 March 2020 to 31 August 2020), the 2nd wave (from 1 September 2020 to 31 December 2020), and the 3rd wave (from 1 January 2021 to 31 August 2021). The epidemic waves were defined based on the peaks of infection of the epidemiological curve observed in the Puglia region [16].

HCWs were classified into doctors (including residents), nurses, technicians, administrative staff, and other HCWs (including biologists, psychologists, and other healthcare professionals). The correct use of standard personal protective equipment (PPE) was adopted as an essential factor for the prevention of SARS-CoV-2 infection. A preventive protocol was applied for all 6030 HCWs working at the University Hospital of Bari and was based on the latest guidelines published by the CDC on the prevention and control recommendations for HCWs during the coronavirus disease [17]. A reporting system was activated to collect and monitor all the close contacts of HCWs with suspected or confirmed SARS-CoV-2 cases. Reports originated with an HCW who had a close contact with a COVID-19 case who alerted a central control room, known as the ‘COVID-19 Control Room’ and the Operative Unit of Occupational Medicine. During the 1st wave, a swab RT–PCR test was performed for close contacts with COVID-19 cases and for symptomatic workers. Moreover, HCWs who had close contact with a confirmed COVID-19 case and with incorrect use of PPE were suspended from work and put in home isolation for a 7-day period. HCWs who tested positive were not allowed to return to work until they were clinically and virologically cured; the latter was defined as negative results on two molecular tests in a 24 h period. If an HCW tested negative, they were allowed to return to work but were subjected to active surveillance to detect early symptoms. All HCWs who had close contact with a confirmed COVID-19 case with correct use of PPE were reported to the COVID-19 Control Room, but they were allowed to continue to work if the swab RT–PCR test was negative. A swab RT–PCR test was performed if workers became symptomatic. During the 2nd and 3rd waves, all asymptomatic HCWs were screened every 14 days for SARS-CoV-2 infection using a nasopharyngeal RT–PCR swab test. Fast-track access to molecular testing was ensured for close contacts with COVID-19 cases and for HCWs with signs and symptoms of COVID-19 (as for the 1st wave). To allow a return to work, the protocol required only one negative nasopharyngeal RT–PCR swab followed by a medical examination to assess the HCW’s health status, focusing on the absence of symptoms related to SARS-CoV-2 infection. The return to work was allowed only to completely asymptomatic HCWs with no signs of the disease. Fast-track access to molecular testing was ensured for HCWs with signs and symptoms of COVID-19 (fever, cough, ageusia, etc.).

A risk assessment for operative units was also performed, identifying “low risk of infection units” (LRIUs) and “high risk of infection units” (HRIUs). The HRIUs are the operative units where COVID-19 patients are treated, where emergency aerosol-generating procedures are performed, and where biological samples are handled (i.e., intensive care unit, infectious disease unit, emergency room, department of microbiology, and virology). During the 2nd and 3rd waves, HRIU workers were screened for SARS-CoV-2 infection more frequently (every 7–10 days) than LRIU workers.

Nasopharyngeal and oropharyngeal swabs were collected and stored in a sterile tube and analyzed in the Hospital Virology Laboratory. Diagnostic testing for SARS-CoV-2 and specimen collection were carried out following CDC guidelines [18]. All the selected workers were submitted to a collection of nasopharyngeal swab specimens by trained staff following adequate standard operating procedures. RT–PCR targeting SARS-CoV-2 RNA was performed based on the detection of unique sequences of viral RNA by real-time reverse transcription-polymerase chain reaction.

The date 27 December 2020 marked the start of the vaccination campaign for HCWs, and 98% of the study population was fully vaccinated with two doses of BNT162b2 mRNA COVID-19 vaccine during the 3rd wave (January 2021–31 August 2021) [19].

All subjects were informed that data from the research protocol would be treated in an anonymous and collective way with scientific methods and for scientific purposes in accordance with the principles of the Declaration of Helsinki. Ethical approval was not necessary because all medical and instrumental examinations were performed according to Italian law concerning the protection of workers exposed to occupational risks (D.Lgs. 81/2008).

### 2.2. Statistical Analysis

The variables considered were all categorical; therefore, the results were expressed as absolute frequencies and percentages. Comparisons among groups of interest were formally conducted with the chi-square test. A *p* value < 0.05 was considered statistically significant. Univariate logistic regression analysis was used to calculate the odds ratio (O.R.) and its confidence interval (C.I.). Data were analyzed using IBM SPSS Statistics Version 26 (SPSS Inc., Chicago, IL, USA, 2019).

## 3. Results

The distribution of COVID-19 cases among HCWs is presented in Table 1. From March 2020 to August 2021, 519 (9%) HCWs tested positive among a total of 6030 HCWs during the three waves. Among these, 34 tested positive during the 1st wave (7% of the overall COVID-19 cases during the three waves and 0.56% of the total of 6030 HCWs), 326 tested positive during the 2nd wave (63% of the overall COVID-19 cases during the three waves and 5.4% of the total of 6030 HCWs), and 159 tested positive during the 3rd wave (30% of the overall COVID-19 cases during the three waves and 4.4% of the total of 6030 HCWs). The highest frequency of COVID-19 cases was observed during the 2nd wave, from 1 September 2020 to 31 December 2020, and the lowest frequency was observed during the 1st wave, from 12 March 2020 to 31 August 2020 (*p* < 0.001). None of the HCWs died from COVID-19. We did not observe any reinfection in the three waves considered. As for the SARS-CoV-2 variants of concern, no variants were analyzed during the first two waves, while during the 3rd wave, 19 cases of B.1.1.7 (Alpha) and 3 cases of B.1.617.2 (Delta) were detected.

Table 2 summarizes the selected characteristics of the 519 HCWs with a laboratory-confirmed diagnosis of SARS-CoV-2. The following characteristics were considered: sex, job, age, and operating unit classified as “high risk of infection unit” and “low risk of infection unit”.

In all three waves, the positivity rate was higher in female HCWs, although this result was not statistically significant (*p* = 0.224); no differences were detected according to age (Table 2).

The frequency of positive doctors and administrative staff was significantly different among the three waves (doctors: *p* < 0.001; administrative staff: *p* = 0.012), while the frequency of nurses, technicians, and other HCWs was not significantly different. In particular, a statistically significant trend was the decrease in positive cases among doctors in the three periods evaluated in the study (*p* < 0.001).

The frequency of positives in the three waves was also compared among all groups of workers and was found to be statistically significant (*p* = 0.001).

During the 1st wave, doctors were the most affected HCWs, while in the 2nd and 3rd waves, nurses were the most affected. In particular, in the 1st wave, 20 of 34 SARS-CoV-2-positive HCWs were doctors (59%) and 11 (32%) were nurses; in the 2nd wave, 99 of 326 SARS-CoV-2-positive HCWs were doctors (30%), 131 (40%) were nurses, 13 (4%) were technicians, and 24 (7%) were administrative workers; in the 3rd wave, 38 of 159 SARS-CoV-2-positive HCWs were doctors (24%), 60 (38%) were nurses, 11 (7%) were technicians, and 22 (14%) were administrative workers.

In none of the 3 waves did working in the HRIU department lead to an increased risk of contracting COVID-19 (1st wave O.R. 1.0, C.I. 0.4-2.3; 2nd wave O.R. 0.98, C.I. 0.7-1.3; and 3rd wave O.R. 0.7, C.I. 0.4-1.1). In all three waves, HCWs in LRIUs showed a higher frequency of COVID-19 cases than HCWs in HRIUs. In detail, 422 HCWs tested positive among 4805 HCWs employed in LRIUs (8.7%), and 97 HCWs tested positive among 1225 HCWs working in HRIUs (7.9%). This difference was not statistically significant (*p* = 0.390).

Finally, a comparison was made between the frequency of COVID-19 among HCWs of the University Hospital of Bari and the frequency observed in the general population of the Puglia region during the three waves [20,21]. In the 1st wave (from 12 March 2020 to 31 August 2020), the frequency of COVID-19 was 0.56% in HCWs of the University Hospital and 1.7% in the general population. During the 2nd wave (from 1 September 2020 to 31 December 2020), the frequency of COVID-19 was 5.4% in HCWs of the University Hospital and 11.5% in the general population. Finally, in the 3rd wave (from 1 January 2021 to 31 August 2021), the frequency of COVID-19 was 4.4% in HCWs of the University Hospital and 7.7% in the general population (Table 3).

## 4. Discussion

The overall frequency of SARS-CoV-2 infection among HCWs of the University Hospital of Bari was very low (9%) in an observation period of 537 days. Careful compliance with correct PPE utilization and biological risk stratification was helpful to avoid nosocomial clusters, keeping high-risk workers in home isolation as soon as possible after hazardous contact and before RT–PCR testing could detect a viral genome. The very low infection rate discovered among exposed HCWs in our protocol supported this hypothesis. Few studies have been published on the evolution of SARS-CoV-2 infection among European HCWs during the 1st, 2nd, and 3rd waves. Recent studies in the Netherlands and UK have reported prevalence rates in HCWs of 9% and 18%, respectively. However, both studies were performed in general hospital settings using only molecular tests on symptomatic HCWs [22,23]. Our result are also in agreement with recent studies comparing HCWs and positivity incidences in patients proving the efficacy of prevention measures adopted by HCWs [24,25,26]. It is also important to point out that few studies have been published regarding HCWs working in other healthcare facilities. The results of these studies show that preventive measures and the use of PPE seem to have effectively protected dentists from contagion, while the situation in terms of infections and stress is more critical for the HCWs of nursing homes [27,28].

We observed a significant impact of the COVID-19 pandemic among HCWs at the University Hospital of Bari during the second epidemic wave (63% of the total number of positive cases). A reasonable explanation is that the rise in the infection rate among HCWs seems to have reflected the increasing spread of SARS-CoV-2 among the overall population of the Puglia region in the same period, as evidenced by other recent studies on HCWs in Italy [29]. Moreover, during the 2nd wave, lockdown measures were less severe throughout the national territory, and there was a wider possibility of regional/national mobility than in the 1st wave. Finally, it cannot be excluded that during the 2nd wave, a lower adherence to prevention measures was observed by HCWs in view of the oncoming vaccination campaign and the greater sense of safety it provided.

During the third epidemic wave, we observed a reduction in the incidence of COVID-19 cases among HCWs following the start of the vaccination campaign in this period [30,31]. We also found a higher frequency of COVID-19 cases among female HCWs. This finding is in accordance with national-level findings [2]. Although the overall absolute number of COVID-19 cases among HCWs increased across all occupational categories during the second wave, the positivity rate for doctors decreased from 59% in the first wave to 30% in the second wave and to 24% in the third wave. This result can be explained by a more careful adherence to the prevention and protection measures implemented by the Operative Unit of Occupational Medicine by doctors compared to other professional groups due to different cultural and professional backgrounds [32,33,34].

Working in a COVID-19 designated or in an HRIU department does not increase the risk of contracting SARS-CoV-2 infection: in all three waves, HCWs in LRIUs showed a nonsignificant higher frequency of COVID-19 cases than HCWs in HRIUs (8.7% vs. 7.9%). This finding, in line with recent studies, confirms the effectiveness of the use of adequate PPE in departments dedicated to the assistance and care of patients affected by COVID-19 or at a high biological risk, such as departments where invasive and aerosol-generating procedures are carried out [35,36,37].

Finally, the comparison of the frequency of COVID-19 cases in HCWs of the University Hospital and the general population of the Puglia region shows that the frequency was always lower among HCWs compared to the general population of the same geographical region, despite the higher biological risk of the hospital setting. However, in analyzing this result, all the limitations and biases that this kind of comparison implies must be considered. This finding is in accordance with recent scientific studies, which show a higher SARS-CoV-2 transmission rate in family settings than in occupational settings, and it also shows the effectiveness and importance of prevention and protection measures and protocols adopted by the hospital in containing the spread of the virus [38]. The low infection rate among HCWs also indirectly points out that HCWs adopted correct anticontagion behaviors in the community outside the hospital, probably due to the sense of responsibility originating from the awareness of their role and from facing the consequences of the COVID-19 disease every day. All HCWs also took a training course on biological risk, in accordance with Italian legislation.

Our study suffers from several limitations. First, the differences in the prevention protocol and in the testing criteria during the three waves may have affected the results, acting as a confounding factor in the frequency quantification of the positive cases. Second, the contribution of vaccination in reducing COVID-19 cases during the third wave was not quantified, and information on SARS-CoV-2 infection status among the contacts of HCWs was not available. Finally, unlike other studies on the same population, no information was available on the viral load of positive tests and comorbidities. Despite this limitation, our study is one of the few to have examined COVID-19 waves in a large population of HCWs that was heterogeneous in terms of jobs for a long period of time since the start of the pandemic.

## 5. Conclusions

In conclusion, our study shows the importance of studying SARS-CoV-2 infection in HCWs with potentially high exposure to the virus. The prevention and protection protocol adopted by the University Hospital of Bari has shown good results, with a low prevalence of COVID-19 cases among HCWs. The correct use of PPE and the early identification of symptomatic workers through strict prevention protocols are still essential factors to avoid nosocomial clusters and, consequently, to protect the health of HCWs and frail patients, even in this current phase of vaccine availability.

## Figures and Tables

**Table 1:** Distribution of Laboratory-Confirmed Diagnoses of SARS-CoV-2 Infections in the Three Waves.

	Period	Positive HCWs		Chi-Square Test
		*n*	%	(*p* Value)
**1st wave** **(6814 NST)**	12/03/20–31/08/20	34	7%	<0.001 *
**2nd wave** **(41780 NST)**	01/09/20–31/12/20	326	63%	
**3rd wave** **(83629 NST)**	01/01/21–31/08/21	159	30%	
	Total	519	100%	

* *p*-value refers to the difference in the frequency of positive HCWs in the three waves (1st wave vs. 2nd wave vs. 3rd wave).

**Table 2:** Selected Characteristics of the 519 HCWs with a Laboratory-Confirmed Diagnosis of SARS-CoV-2 Infection.

	1st Wave March–August 2020		2nd Wave September–December 2020		3rd Wave January–August 2021		Chi-Square Test (*p*-Value) *
**Sex**	***n***	**%**	***n***	**%**	***n***	**%**	0.224
**Female**	24	71%	183	56%	97	61%	
**Male**	10	29%	143	44%	62	39%	
**Total**	34	100%	326	100%	159	100%	
**Job title**	***n***	**%**	***n***	**%**	***n***	**%**	
**Doctors**	20	59%	99	30%	38	24%	<0.001
**Nurses**	11	32%	131	40%	60	38%	0.602
**Technicians**	1	3%	13	4%	11	7%	0.160
**Administrative**	0	0%	24	7%	22	14%	0.012
**Other HCWs**	2	6%	59	18%	28	18%	0.194
**Total**	34	100%	326	100%	159	100%	
**Age group**	***n***	**%**	***n***	**%**	***n***	**%**	
**20–29**	6	18%	52	16%	28	18%	0.903
**30–39**	9	26%	61	19%	27	17%	0.427
**40–49**	7	21%	62	19%	32	20%	0.876
**50–59**	8	24%	101	31%	50	31%	0.649
**60–69**	4	12%	50	15%	22	14%	0.790
**Total**	34	100%	326	100%	159	100%	
**Risk**	***n***	**%**	***n***	**%**	***n***	**%**	0.390
**High risk of infection Unit**	7	21%	66	20%	24	15%	
**Low risk of infection Unit**	27	79%	260	80%	135	85%	
**Total**	34	100%	326	100%	159	100%	

* *p*-value refers to the significance of the difference in the frequency of positives among columns.

**Table 3:** Frequency of SARS-CoV-2 Infection between HCWs and the General Population of the Puglia Region.

	SARS-CoV-2 INFECTION CASES (%)	
	HCWs (*n*. 6030)	GENERAL POPULATION (*n*. 3912, 166)
**1st wave** 12 March 2020–31 August 2020	0.56%	1.7%
**2nd wave** 1 September 2020–31 December 2020	5.4%	11.5%
**3rd wave** 1 January 2021–31 August 2021	4.4%	7.7%

## Data Availability

The data presented in this study are available on request from the corresponding author.

## References

[1] Armocida B., Formenti B., Ussai S., Palestra F., Missoni E. (2020). The Italian health system and the COVID-19 challenge. Lancet Public Health.

[2] Italian Higher Institute of Health (ISS) https://www.epicentro.iss.it/coronavirus/sars-cov-2-dashboard.

[3] The Lancet (2020). COVID-19: Protecting health-care workers. Lancet.

[4] Barranco R., Ventura F. (2020). COVID-19 and infection in health-care workers: An emerging problem. Med. Leg. J..

[5] Guarnieri V., Moriondo M., Giovannini M., Lodi L., Ricci S., Pisano L., Barbacci P., Bini C., Indolfi G., Zanobini A. (2021). Surveillance on Healthcare Workers during the First Wave of SARS-CoV-2 Pandemic in Italy: The Experience of a Tertiary Care Pediatric Hospital. Front. Public Health.

[6] Wee L.E., Sim X.Y.J., Conceicao E.P., Aung M.K., Goh J.Q., Yeo D.W.T., Gan W.H., Chua Y.Y., Wijaya L., Tan T.T. (2020). Containment of COVID-19 cases among healthcare workers: The role of surveillance, early detection, and outbreak management. Infect. Control Hosp. Epidemiol..

[7] Haque M., Sartelli M., McKimm J., Abu Bakar M. (2018). Health care-associated infections-An overview. Infect. Drug Resist..

[8] Wee L.E., Fua T.P., Chua Y.Y., Ho A.F.W., Sim X.Y.J., Conceicao E.P., Venkatachalam I., Tan K.B., Tan B.H. (2020). Containing COVID-19 in the Emergency Department: The Role of Improved Case Detection and Segregation of Suspect Cases. Acad. Emerg. Med..

[9] European Centre for Disease Prevention and Control (ECDC) Infection Prevention and Control and Preparedness for COVID-19 in Healthcare Settimgs. Sixth Update–9 February 2021. https://www.ecdc.europa.eu/sites/default/files/documents/Infection-prevention-and-control-in-healthcare-settings-COVID-19_6th_update_9_Feb_2021.pdf.

[10] World Health Organization (WHO) https://www.who.int/publications/i/item/WHO-2019-nCoV-clinical-2021-2.

[11] Centers for Disease Control and Prevention (CDC) https://www.cdc.gov/coronavirus/2019-ncov/hcp/guidance-risk-assesment-hcp.html.

[12] Vimercati L., De Maria L., Quarato M., Caputi A., Stefanizzi P., Gesualdo L., Migliore G., Fucilli F.I.M., Cavone D., Delfino M.C. (2021). COVID-19 hospital outbreaks: Protecting healthcare workers to protect frail patients. An Italian observational cohort study. Int. J. Infect. Dis..

[13] Vimercati L., Dell’Erba A., Migliore G., De Maria L., Caputi A., Quarato M., Stefanizzi P., Cavone D., Ferorelli D., Sponselli S. (2020). Prevention and protection measures of healthcare workers exposed to SARS-CoV-2 in a university hospital in Bari, Apulia, Southern Italy. J. Hosp. Infect..

[14] Boffetta P., Violante F., Durando P., De Palma G., Pira E., Vimercati L., Cristaudo A., Icardi G., Sala E., Coggiola M. (2021). Determinants of SARS-CoV-2 infection in Italian healthcare workers: A multicenter study. Sci. Rep..

[15] Vimercati L., Stefanizzi P., De Maria L., Caputi A., Cavone D., Quarato M., Gesualdo L., Lopalco P.L., Migliore G., Sponselli S. (2021). Large-scale IgM and IgG SARS-CoV-2 serological screening among healthcare workers with a low infection prevalence based on nasopharyngeal swab tests in an Italian university hospital: Perspectives for public health. Environ. Res..

[16] https://statistichecoronavirus.it/coronavirus-italia/coronavirus-puglia/.

[17] Centers for Disease Control and Prevention (CDC) https://www.cdc.gov/coronavirus/2019-ncov/hcp/infection-control-recommendations.html.

[18] Centers for Disease Control and Prevention Interim Guidelines for Collecting, Handling, and Testing Clinical Specimens from Persons for Coronavirus Disease 2019 (COVID-19) Published online 2020. https://www.cdc.gov/coronavirus/2019-ncov/lab/guidelines-clinical-specimens.html.

[19] Bianchi F.P., Germinario C.A., Migliore G., Vimercati L., Martinelli A., Lobifaro A., Tafuri S., Stefanizzi P., Control Room Working Group (2021). BNT162b2 mRNA COVID-19 Vaccine Effectiveness in the Prevention of SARS-CoV-2 Infection: A Preliminary Report. J. Infect. Dis..

[20] Statistical Office Puglia Region. https://www.regione.puglia.it/web/ufficio-statistico/andamento-dei-contagi.

[21] Epidemiological Bulletins Puglia Region. https://regione.puglia.it/web/speciale-coronavirus/elenco-bollettini-covid.

[22] Tostmann A., Bradley J., Bousema T., Yiek W.K., Holwerda M., Bleeker-Rovers C., Ten Oever J., Meijer C., Rahamat-Langendoen J., Hopman J. (2020). Strong associations and moderate predictive value of early symptoms for SARS-CoV-2 test positivity among healthcare workers, the Netherlands, March 2020. Eurosurveill.

[23] Keeley A.J., Evans C., Colton H., Ankcorn M., Cope A., State A., Bennett T., Giri P., de Silva T.I., Raza M. (2020). Roll-out of SARS-CoV-2 testing for healthcare workers at a large NHS Foundation Trust in the United Kingdom, March 2020. Eurosurveill.

[24] Cattelan A.M., Sasset L., Di Meco E., Cocchio S., Barbaro F., Cavinato S., Gardin S., Carretta G., Donato D., Crisanti A. (2020). An Integrated Strategy for the Prevention of SARS-CoV-2 Infection in Healthcare Workers: A Prospective Observational Study. Int. J. Environ. Res. Public Health.

[25] Tong X., Ning M., Huang R., Jia B., Yan X., Xiong Y., Wu W., Liu J., Chen Y., Wu C. (2020). Surveillance of SARS-CoV-2 infection among frontline health care workers in Wuhan during COVID-19 outbreak. Immun. Inflamm. Dis..

[26] Suzuki T., Hayakawa K., Ainai A., Iwata-Yoshikawa N., Sano K., Nagata N., Suzuki T., Wakimoto Y., Akiyama Y., Miyazato Y. (2021). Effectiveness of personal protective equipment in preventing severe acute respiratory syndrome coronavirus 2 infection among healthcare workers. J. Infect. Chemother..

[27] Halcomb E., McInnes S., Williams A., Ashley C., James S., Fernandez R., Stephen C., Calma K. (2020). The Experiences of Primary Healthcare Nurses during the COVID-19 Pandemic in Australia. J. Nurs. Scholarsh..

[28] Kim D., Ko J.H., Peck K.R., Baek J.Y., Moon H.W., Ki H.K., Yoon J.H., Kim H.J., Choi J.H., Park G.E. (2021). A COVID-19 Exposure at a Dental Clinic Where Healthcare Workers Routinely Use Particulate Filtering Respirators. Int. J. Environ. Res. Public Health.

[29] Comelli A., Consonni D., Lombardi A., Viero G., Oggioni M., Bono P., Uceda Renteria S.C., Ceriotti F., Mangioni D., Muscatello A. (2021). Nasopharyngeal Testing among Healthcare Workers (HCWs) of a Large University Hospital in Milan, Italy during Two Epidemic Waves of COVID-19. Int. J. Environ. Res. Public Health.

[30] Haviari S., Bénet T., Saadatian-Elahi M., André P., Loulergue P., Vanhems P. (2015). Vaccination of healthcare workers: A review. Hum. Vaccin Immunother.

[31] Prato S., Paladino M.E., Riva M.A., Belingheri M. (2021). COVID-19 Vaccination and Asymptomatic Infection: Effect of BNT162b2 mRNA Vaccine on the Incidence of COVID-19 and Duration of Sick Leave Among Healthcare Workers. J. Occup. Environ. Med..

[32] Oksanen L.A., Sanmark E., Oksanen S.A., Anttila V., Paterno J.J., Lappalainen M., Lehtonen L., Geneid A. (2021). Sources of healthcare workers’ COVID-19 infections and related safety guidelines. Int. J. Occup. Med. Environ. Health.

[33] World Health Organization (WHO) Coronavirus Disease (COVID-19) Outbreak: Rights, Roles and Responsibilities of Health Workers, Including Key Considerations for Occupational Safety and Health. https://www.who.int/publications/i/item/WHO-2019-nCoV-HCW_advice-2021-1.

[34] Shah A.S., Wood R., Gribben C., Caldwell D., Bishop J., Weir A., McAllister D.A. (2020). Risk of hospital admission with coronavirus disease 2019 in healthcare workers and their households: Nationwide linkage cohort study. BMJ.

[35] Emecen A.N., Keskin S., Boncukcu Eren E., Yildirim Ustuner B., Celik S.G., Suner A.F., Sezgin E., Siyve N., Basoglu Sensoy E., Tutal Altas E. (2022). Impact of social contacts on SARS-CoV-2 exposure among healthcare workers. Occup. Med..

[36] Zhou P., Huang Z., Xiao Y., Huang X., Fan X.G. (2020). Protecting Chinese healthcare workers while combating the 2019 novel coronavirus. Infect. Control Hosp. Epidemiol..

[37] Ran L., Chen X., Wang Y., Wu W., Zhang L., Tan X. (2020). Risk Factors of Healthcare Workers With Coronavirus Disease 2019: A Retrospective Cohort Study in a Designated Hospital of Wuhan in China. Clin. Infect. Dis..

[38] Thompson H.A., Mousa A., Dighe A., Fu H., Arnedo-Pena A., Barrett P., Ferguson N.M. (2021). Severe Acute Respiratory Syndrome Coronavirus 2 (SARS-CoV-2) Setting-specific Transmission Rates: A Systematic Review and Meta-analysis. Clin. Infect. Dis..

